# Structural brain correlates of balance control in children with cerebral palsy: baseline correlations and effects of training

**DOI:** 10.1007/s00429-025-02937-1

**Published:** 2025-05-20

**Authors:** Nina P. T. Jacobs, Marjolein M. van der Krogt, Annemieke I. Buizer, Laura A. van de Pol, Chloé E. C. Bras, Frederik Barkhof, Pieter Meyns, Petra J. W. Pouwels

**Affiliations:** 1 Department of Rehabilitation Medicine, Amsterdam UMC, Vrije Universiteit, Amsterdam, The Netherlands; 2 Amsterdam Movement Sciences, Rehabilitation & Development, Amsterdam, The Netherlands; 3 Emma Children’s Hospital, Amsterdam UMC, Amsterdam, The Netherlands; 4 Department of Child Neurology, Amsterdam UMC, Vrije Universiteit, Amsterdam Neuroscience, Amsterdam, The Netherlands; 5 Department of Radiology & Nuclear Medicine, Amsterdam UMC, Vrije Universiteit, Amsterdam Neuroscience, Amsterdam, The Netherlands; 6 UCL Institutes of Neurology and Healthcare Engineering, London, UK; 7 REVAL Rehabilitation Research, Faculty of Rehabilitation Sciences, Hasselt University, Diepenbeek, Belgium

**Keywords:** Cerebral palsy, Neuroplasticity, Postural balance, Rehabilitation, Magnetic resonance imaging, Diffusion tensor based morphometry

## Abstract

Structural brain abnormalities likely underlie impaired balance control in cerebral palsy (CP). This study investigated whether balance measures were associated with measures derived from conventional MRI and diffusion tensor imaging (DTI), and whether an X-Box One Kinect balance training (6 weeks, 5 days/week, 30 min/session) could induce neuroplastic changes in CP. Twelve children with spastic CP (age:11.3 ± 2.3y) underwent balance evaluation and MRI examination, at baseline and after training. Nine age-matched typically developing (TD) children underwent baseline measurements. Balance control was evaluated testing advanced motor skills (Challenge score) and during gait (medio-lateral Margin of Stability, MoS). With conventional MRI, but especially with DTBM (DTI-based VBM), we found smaller volumes of several deep grey matter structures and within the right inferior parietal cortex, right supramarginal cortex, and left postcentral cortex, and lower fractional anisotropy (FA) and smaller volumes of various white matter regions in CP compared to TD. Within the CP group alone, no correlations within brain tissue were found. After training, Challenge scores of children with CP improved. In an exploratory analysis DTBM showed a trend for volume increase within the right inferior parietal cortex, volume decrease within the right retrolenticular limb of the internal capsule, and an increase of FA within the right corticospinal tract. This indicates that a 6-week balance intervention may induce neuroplastic changes in children with CP.

CP-RehOP (trial registration number: NTR6034/NL5854, date of registration: August 26th 2016).

**Supplementary Information:**

The online version contains supplementary material available at 10.1007/s00429-025-02937-1.

## Introduction

Cerebral palsy (CP) is an umbrella term that describes a heterogeneous group of movement deficits, caused by brain malformation or non-progressive brain damage in developing fetuses or infants (Bax et al. 2005). Typical CP-related motor problems include spasticity, lower selective motor control and muscle weakness, which often result in impaired balance control (Pavão et al. 2013). To improve children’s gross motor skills, and thereby boost their independent mobility and activities, improving balance control is a common goal of interventions in CP (Woollacott and Shumway-Cook 2005). The main treatment for impaired balance control is physical therapy, often in combination with more general gross motor training, or combined with other adjuvant therapies (e.g. hippotherapy, orthotic devices) (Araújo et al. 2020). However, in some cases orthoses may have a possibly negative effect on balance control during gait (Meyns et al. 2020). A lack of accessible treatment interventions with proven, significant effects on balance control is apparent for children with CP.

Various factors contribute to impaired balance control in CP. In general, balance control is determined by musculoskeletal factors (e.g. muscle strength and posture), and neural factors (sensory integration and neuromotor control) (Woollacott and Burtner 1996; Woollacott and Shumway-Cook 1990). Hence, in CP, both musculoskeletal impairments, such as muscle weakness or altered musculoskeletal alignment, and neural impairments, such as spasticity or increased co-activation, can contribute to poor balance control (Woollacott and Burtner 1996; Woollacott and Shumway-Cook 2005). Research in healthy participants and a range of patient populations suggested that several brain structures, such as the cerebellum, basal ganglia, thalamus, hippocampus and inferior parietal cortex, are likely involved in balance control (Surgent et al. 2019). Yet, it is not known whether this information can be inferred to children with CP, as they often have lesions in these regions (Scheck et al. 2014), and brain structural and functional connectivity may be altered (Jacobs et al. 2023). To date, however, the relation between CP-related alterations in brain structure and balance control has been investigated minimally. A recent study described a significant relation between CP-related alterations in brain structure and balance control (Jaatela et al. 2023). This study, however, focused solely on the white matter integrity of the corpus callosum and the transcallosal fiber bundles, whereas it may be expected that other white matter tracts and brain volumetric regions can be related to balance control as well.

Conventional magnetic resonance imaging (MRI) and diffusion tensor imaging (DTI) have been used previously to investigate the structural brain correlates of impaired balance control in traumatic brain injury (TBI) patients. In TBI patients balance deficits result from brain injury. In TBI patients, infratentorial white matter (WM) and grey matter (GM) loss, as assessed with conventional MRI, and lower fractional anisotropy (FA) of several WM regions (e.g. middle and superior cerebellar peduncle), as assessed with DTI, have been associated with impaired balance control (Caeyenberghs et al. 2010; Drijkoningen et al. 2015b). Regional brain volume loss (e.g. in thalamus or basal ganglia) and WM abnormalities (e.g. lower FA in internal capsule or corpus callosum) are also common in CP (Yoshida et al. 2011; Scheck et al. 2014; Arrigoni et al. 2016). Alterations in brain structure may therefore underlie impaired balance control in CP as well. However, results of TBI should not be directly extended to CP, because TBI patients most often have had many years of typical brain development, whereas brain injury in CP occurred in early stages of brain development. Recently, volume loss and lower FA in the corpus callosum and in sensorimotor tracts have been observed in adolescents with cerebral palsy compared to typically developing participants, and associations with stability were observed within the whole cohort (Jaatela et al. 2023). This shows the importance of studying the structural brain correlates of impaired balance control in CP. By linking structural abnormalities to balance deficits, clinicians can better understand why a child has more severe postural control problems than others. In the future, this could give insights into more individualized treatment possibilities based on a person’s structural brain abnormalities, and possibly predict therapy outcome.

Secondly, although it is known that balance interventions can have positive effects in children with CP (Kim and Lee 2013; Dewar et al. 2015), the structural brain correlates of training-induced improvement in balance control remain poorly understood. It is believed that training interventions generally have the potential to induce neuroplastic changes in individuals with CP (Kurz et al. 2012; Sterling et al. 2013; Bleyenheuft et al. 2015). However, the effect of a balance control intervention on structural neuroplastic changes in CP has been explored only by Rasooli et al, (2017). This pilot study of four participants described that improvement in balance control, after two months of anti-gravity treadmill training, was accompanied by increases in FA of infratentorial WM, as assessed with DTI. In TBI patients it has been found that improvement in balance control, after eight weeks of balance training, was associated with an increase of mean diffusivity of the inferior cerebellar peduncle, as assessed with DTI (Drijkoningen et al. 2015a). Thus, first results suggest that balance training interventions can induce neuroplastic changes in brain structure, but more research is needed to confirm this hypothesis in children with CP.

Commercial virtual reality (VR) games are increasingly used for motor training and to improve balance (Truijen et al. 2022; Xue et al. 2025). These games are of particular interest to pediatric populations, such as children with CP, as they focus on joy and motivation, and are a powerful medium to provide feedback about a person’s movements. VR games for rehabilitation to train balance in CP have been shown to be effective (Liu et al. 2022; Meyns et al. 2022; Komariah et al. 2024). For specific populations including children, non-immersive VR training has an advantage of not requiring a (heavy) headset, showing promising effects as well (Bieryla 2016; Wang et al. 2023). Such non-immersive gamified interventions may have potential for broader accessibility in children with CP, especially if the training promotes balance control and neuroplasticity.

Our overall aim was to improve our understanding of the structural brain correlates of balance control in CP. This can ultimately lead to development of prognostic markers and/or balance interventions. Our first objective was to gain insight in CP-related alterations in balance control and brain structure, by investigating differences between children with CP and typically developing (TD) children concerning balance measures, conventional MRI-derived brain volume and cortical thickness measures, and voxel-wise FA and volume measures obtained with DTI-driven tensor based morphometry (DTBM) (Zhang et al. 2007; Keihaninejad et al. 2013; Dennis et al. 2016; Lee et al. 2020). The areas with differences between CP and TD were used as regions-of-interest (ROIs) for the subsequent objectives. Our second objective was to investigate possible associations between impaired balance control and altered brain structure in children with CP, by investigating correlations between balance measures and DTBM measures, for all children and separately within the CP group. Finally, our third objective was to explore whether anticipated training-induced improvement in balance control was accompanied by changes in brain structure, by investigating whether a 6-week X-Box One Kinect balance training could induce neuroplastic changes in DTBM measures, in children with CP.

## Methods

### Study outline

The study was conducted as a part of the registered trial CP-RehOP (trial number: NTR6034/NL5854). More information about the goals of this trial can be found in the supplementary material. For this exploratory study, a convenience sample from the Amsterdam UMC outpatient clinic was included. The recruitment period was constrained to two years, restricted by project finances. Children with CP and TD children were included, and all children underwent baseline balance control and MRI examinations. Subsequently, children with CP participated in a 6-week balance intervention, after which they had follow-up balance control and MRI examinations.

### Participants

A total of 12 children with CP and 9 TD children participated in this study. For children with CP, the following inclusion criteria were applied: (1) clinical diagnosis of bilateral spastic CP, (2) current classification as level II on the Gross Motor Function Classification System (GMFCS) (Palisano et al. 1997), indicating difficulty walking long distances and balancing, (3) presence of brain abnormalities on conventional MRI matching a diagnosis of CP, as confirmed by the neuroradiologist, (4) cognitive abilities enabling basic communication. Exclusion criteria for children with CP were: (1) presence of neurologic diseases other than CP, (2) orthopedic surgery or selective dorsal rhizotomy in the last 12 months, (3) botulinum toxin injections in the last six months. Exclusion criteria for TD children were: (1) known neural and/or orthopedic abnormalities, (2) presence of brain abnormalities on conventional MRI.

Prior to participation, informed consent was obtained from all parents and from all children who were 12 years or older. Ethical approval was obtained from the Medical Ethics Committee of VU University Medical Center Amsterdam.

### Balance intervention

Children with CP participated in a home-based balance training, using the X-Box One Kinect (Microsoft), and practiced for 6 weeks, 5 days/week and 30 min/session. During training sessions, children played tennis, soccer, and bowling subgames of Kinect Sports Rivals (Rare Ltd.), during which the body of the children functioned as the controller. Since tennis required arm swing and trunk rotation, soccer demanded standing on one leg, and bowling needed arm swinging while doing a lunge, balance was challenged throughout the games, and balance control was expected to improve post-training. Time played on the X-Box on the children’s personal account was automatically saved and was read by the researchers to estimate whether the imposed training intensity (15 h) was achieved.

### Balance measures

Two balance measures were obtained: maximal score on the Challenge module of the Gross Motor Function Measure (GMFM) and medio-lateral margin of stability (MoS) during gait.

The Challenge module, developed supplementary to the conventional GMFM, is used to evaluate gross motor abilities of children with CP who have relatively good motor skills (Wilson et al. 2011; Glazebrook and Wright 2014; Sargent and Fetters 2014). It has excellent inter-rater and test–retest reliability (Lam et al. 2015) and its concurrent validity of high-level gross motor assessment batteries has been established (Clutterbuck et al. 2021). In the Challenge module, performance during 20 advanced gross-motor tasks, focusing on balance control, speed and coordination, is scored on a 0–4 ordinal scale (best of three trials), thereby integrating quality and speed of performance (Wilson et al. 2011). Example items are standing on one leg, walking on a balance beam, throwing and catching of a basketball, and waving through pylons. Scores on all individual items were summed to obtain the Challenge score. Higher scores are indicative of better balance control (Wilson et al. 2011). Previous pilot research has shown benefits of VR in children with CP using this measure (Levac et al. 2018).

MoS is a measure of balance control during steady-state gait. A detailed description of MoS with corresponding references is given in the supplementary material. In short, MoS was determined during walking on an instrumented treadmill. Prior to the measurements, body mass, body height and limb lengths were obtained, and a total of 39 retroreflective markers were attached to the children in accordance with the Vicon full body plug-in gait model (as implemented in Vicon Nexus 2.5). Children walked for 6 min at their preferred speed on the treadmill for habituation. Subsequently, children walked at preferred walking speed during one trial of one minute, during which kinetic and kinematic data was collected. The position of the extrapolated centre of mass was calculated to correct for the effects of walking speed on the position of the centre of mass (Hof et al. 2005). MoS was defined as the minimal medio-lateral distance between the extrapolated centre of mass and the lateral malleolus of the leading foot (Hof et al. 2005; Hak et al. 2013). Average MoS was calculated as the average value over all available steps. In children with CP increases in medio-lateral MoS have been reported (e.g. Delabastita et al. 2016; Rethwilm et al 2021; Sangeux et al 2024). Accordingly, lower MoS values are considered indicative of better balance control. MoS has also been applied to determine the effectiveness of ankle foot orthoses in children with CP on balance control during walking (Meyns et al 2020).

### MRI acquisition

MRI data acquisition was performed using a 3T MR scanner, equipped with an 8-channel head coil (GE Discovery MR750), in the VU University Medical Center. Conventional MRI was acquired using 3D sagittal T1-weighted images (TR/TE/TI = 8.2/3.2/450 ms, voxel size = 1 × 1 × 1 mm^3^) and 3D sagittal T2-weighted FLAIR images (TR/TE/TI = 8000/126/2350 ms, voxel size = 1 × 1 × 1.2 mm^3^). Images were corrected for geometrical distortions due to gradient nonlinearity, as implemented on the scanner, prior to further analysis. Diffusion-weighted MRI (DWI) was acquired using 2D echo-planar imaging (TR/TE = 7200/85 ms, voxel size = 2 × 2 × 2 mm^3^, parallel imaging factor 2). 30 volumes were obtained with different gradient directions at a b-value of 1000 s/mm^2^, along with 5 reference volumes at b = 0 s/mm^2^. In addition, two reference scans with reversed phase-encode blips were acquired to correct for echo-planar imaging distortions.

### Conventional MRI processing

Conventional MRI processing was done with tools implemented in FMRIB Software Library (FSL version 5.0.10) (Jenkinson et al. 2012). Since segmentation of T1 images is affected by hypointensities (Chard et al. 2010), and periventricular lesions are common in the CP group, these lesions were detected on FLAIR, linearly registered to T1, and lesion-filled with signal intensity of normal WM, all steps being part of the Lesion Segmentation Toolbox, version 2.0.15 (Schmidt 2017). Volumes of FLAIR hyperintense lesions were also determined.

Then, T1 images were segmented with SIENAX into cerebrospinal fluid and total brain volume, consisting of total WM and total GM (Smith et al. 2002). Subsequently, total WM and total GM volumes were corrected for presence of subcortical structures, which were segmented using FIRST (Patenaude et al. 2011). Volumes of thalamus, basal ganglia (caudate nucleus, globus pallidus and putamen) and hippocampus, were bilaterally summed. Segmentation of the cerebellum was performed by linear and non-linear registration algorithms (FLIRT and FNIRT, respectively) of the Montreal Neurological Institute (MNI) brain atlas (at 25% threshold) from MNI space to T1 subject space. To account for differences in head size, all volumes were spatially normalized for skull size using the scaling parameter determined by SIENAX. Larger scaling parameters indicate smaller heads.

Cortical thickness was determined using the cross-sectional stream (CP and TD at baseline) and longitudinal stream (CP at baseline and follow-up) of FreeSurfer, version 6.0.0 (Dale et al. 1999; Fischl et al. 1999; Reuter et al. 2012). We focused on bilateral precentral gyrus, postcentral gyrus, paracentral gyrus, inferior parietal cortex and parahippocampal gyrus. These regions were depicted based on general associations with motor control and/or the review of Surgent et al. (2019). Cortical thicknesses were averaged across hemispheres, weighted by corresponding inner WM surface area. All segmentations were visually inspected.

### DWI processing

Pre-processing of DWI included estimation and correction of susceptibility-induced distortions, correction of eddy currents and head motion (including detection and imputation of outlier slices), and brain extraction using the FSL tools TOPUP, EDDY, and BET (Andersson et al. 2003, 2016; Andersson and Sotiropoulos 2016). A diffusion tensor model was fitted voxel-wise, using DTIFIT, and the map with sum of squared errors (SSE) was visually checked for artefacts.

Then, a spatially-normalized study-specific tensor group template was created using DTI-TK, which uses an affine and deformable registration algorithm with explicit tensor reorientation optimization (Zhang et al. 2006, 2007). To create a group template with identical weighting of CP and TD participants, and to prevent a bias towards either baseline or follow-up measurements in CP, we followed the pipeline described by (Keihaninejad et al. 2013). First, for all CP participants with 2 time points, a within-subject template was created. Subsequently, a group template was built using the within-subject templates of 8 CP participants, and the baseline images of 1 CP and 9 TD participants. Thus, 3 CP participants were not used to create the group template, but were registered to the template in a next step. Those 3 participants had either extensive tissue loss mainly in the left frontal and parietal lobe (1 subject), or slightly smaller coverage of the cerebellum (2 participants). Then, all participants’ tensors were transformed to the group template, followed by calculation of FA. For participants with 2 time points, mapping from baseline or follow-up native space to that subject’s within-subject template and mapping from the within-subject template to the group template were combined to create the deformation field defining the mapping directly from native space to the group template space (Keihaninejad et al. 2013).

The group template was integrated in all subsequent analyses. To compare local volumetric differences with DTBM, we determined the natural logarithm of the Jacobian determinant field (ln-Jac) within the space of DTI-TK group template (Zhang et al. 2007; Lee et al. 2020). Ln-Jac indicates to what extent a particular structure needs to be inflated or compressed to match the group template, and gives information about the size of that structure in each subject compared to the group template. A smaller structure leads to a negative ln-Jac value for that subject, and a larger structure leads to a positive ln-Jac.

To identify and label brain areas within the DTI-TK group template we used (i) the JHU-ICBM atlas (provided by FSL) for a few selected WM regions (posterior and retrolenticular limb of the internal capsule (PLIC and RLIC), cerebral peduncle, and inferior, middle, and superior cerebellar peduncles); (ii) the Desikan atlas for cortical and deep grey matter (DGM) regions from the IIT Human Brain Atlas (v5.0) (provided by NITRC: NeuroImaging Tools and Resources Collaboration Desikan et al. 2006; Zhang and Arfanakis 2018); (iii) the IIT-WM atlas to determine the most probable tracts running through a selected region (Qi and Arfanakis 2021). These atlases were transformed to the DTI-TK group template using linear and non-linear registrations. The position of the atlases and corresponding ROIs was visually checked for each subject in DTI-TK space to determine the quality of segmentation using DTI-TK registration.

### Statistical analysis, including DTBM

Normality of the data was assessed by the moduli of the Z-scores of skewness and kurtosis, and the Shapiro-Wilks Normality Test. The normality hypothesis was rejected when at least two of these measures indicated that the data was not normally distributed. Homogeneity of variance was assessed using Levene’s test of variance. For demographic data, independent t-tests were used to investigate baseline differences in age, height and body mass between CP and TD, and the Chi-Square test was used to assess group differences in sex. Differences in baseline balance and MRI measures between groups (objective 1) were investigated using independent t-tests. All values are presented as mean ± standard deviation (SD) unless indicated otherwise. These statistical analyses were carried out using IBM SPSS version 26.

Voxel-wise DTBM analyses were performed using FSL’s randomise and 100.000 permutations (Winkler et al. 2014). To define the brain areas (ROIs) for voxel-wise longitudinal and correlation analyses in CP, we first determined voxel-wise differences between CP and TD at baseline regarding FA and/or volume (ln-Jac) (Zhang et al. 2007; Lee et al. 2020). We performed this analysis in a brain mask covering slices present in all participants (thus excluding a few superior and inferior slices from the group template). Voxels with differences between CP and TD based on threshold-free cluster enhancement (TFCE) and family-wise error (FWE) corrected p < 0.05 (regarding FA and/or volume) were combined, binarized, and used as ROI mask for subsequent analyses.

For all participants at baseline, voxel-wise correlations of FA and volume with balance measures were performed within this ROI mask. Subsequently, this correlation analysis was performed for all 12 CP participants at baseline. In these analyses, age was used as covariate. A longitudinal voxel-wise analysis for CP participants was performed using the same ROI mask. Analyses were performed with TFCE and we reported p-values after FWE correction. Due to the explorative design of this study, we also inspected statistical maps which were not corrected for multiple comparisons, as long as corrected p was < 0.5. In this case we addressed TFCE clusters of at least 3 voxels at FWE-uncorrected p < 0.001.

## Results

### Participants

The cohort consisted of 12 children with bilateral spastic CP (GMFCS II) and 9 TD children. Demographic details at baseline are summarized in Table 1. There were no differences between groups regarding sex, age, height and body mass. Primary brain injuries of children with CP are summarized in supplementary Table 1; most children had periventricular leukomalacia. Supplementary Fig. 1 illustrates the varying severity of brain abnormalities. One child with CP was lost at follow-up because of botulinum toxin treatment. Therefore, we could evaluate 11 children with CP at follow-up.

**Table 1:** Demographic details of CP and TD at baseline

	CP (n = 12) (mean ± SD)	TD (n = 9) (mean ± SD)	p
Participants (no.)	12	9	–
Sex (M/F)	8/4	5/4	0.60
Age (year)	11.3 ± 2.3	11.2 ± 2.5	0.91
Height (m)	1.47 ± 0.13	1.55 ± 0.15	0.21
Body mass (kg)	40.9 ± 13.2	43.1 ± 12.5	0.70

*CP* cerebral palsy, *TD* typically developing, *SD* standard deviation

### Data quality

After visual inspection of the conventional MRI-derived volumes or cortical thicknesses, none of the data had to be removed from analysis. For DWI, the median number of imputed slices per subject was 10 (range 0–27), corresponding to less than 0.5%. Furthermore, none of the data was rejected based on visual checks of the SSE maps. Registration from subject space to DTI-TK group template resulted in good alignment of individual DTI images, even in children with CP with clear tissue loss.

### Balance control and brain structure in CP versus TD (objective 1)

Challenge scores in CP were lower, and MoS was higher compared to TD (Table 2). Volumetric analysis of T1 images showed smaller normalized volumes of total WM, thalamus, hippocampus and basal ganglia in CP compared to TD. Volumes of total GM and cerebellum, and measures of cortical thickness did not differ between the groups. Head size scaling parameters were not significantly different between groups. FLAIR hyperintense lesion volumes in CP were not normally distributed, with a median value of 0.96 mL (range 0.02–8.09 mL).

**Table 2:** Balance and conventional MRI measures in CP versus TD

	CP (n = 12) (mean ± SD)	TD (n = 9) (mean ± SD)	p
Balance control			
Challenge score (points)	38.8 ± 12.5	89.6 ± 1.5	**<0.001**
Margin of stability (m)	0.165 ± 0.033	0.135 ± 0.027	**0.05**
Normalized T1-derived brain volumes (mL)			
Total brain	1705 ± 109	1765 ± 43	0.10
Total white matter	623 ± 39	652 ± 14	**0.03**
Total grey matter	1001 ± 79	1019 ± 39	0.52
Thalamus	18.3 ± 1.8	23.0 ± 1.5	**<0.001**
Basal ganglia	28.2 ± 3.8	33.1 ± 1.8	**<0.01**
Hippocampus	9.3 ± 1.5	11.0 ± 0.7	**<0.01**
Cerebellum	215 ± 16	222 ± 17	0.34
Scaling parameter	1.49 ± 0.17	1.39 ± 0.10	0.12
Cortical thickness (mm)			
Precentral gyrus	2.68 ± 0.12	2.77 ± 0.14	0.12
Postcentral gyrus	2.28 ± 0.18	2.29 ± 0.16	0.87
Paracentral gyrus	2.68 ± 0.26	2.74 ± 0.14	0.51
Inferior parietal cortex	2.71 ± 0.13	2.78 ± 0.13	0.30
Parahippocampal gyrus	2.89 ± 0.28	3.11 ± 0.24	0.08
Lesion volume (mL) − median (range)	0.96 (0.02 − 8.09)	–	

Differences in balance and conventional MRI measures between CP and TD at baseline. n = 12 for the CP group. Significant differences (p < 0.05) are shown in bold

*CP* cerebral palsy, *TD* typically developing, *SD* standard deviation

**Fig. 1:**
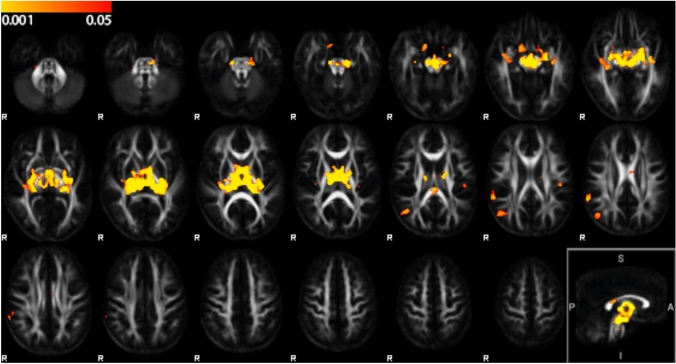
Voxel-wise volumetric comparison (ln-Jac) between CP and TD participants. Yellow–red clusters shown on top of the mean FA group template indicate areas with significantly smaller volumes in CP participants compared to TD participants (TFCE-FWE-corrected p < 0.05, range of p-values indicated by the colorbar). Top row: voxels located in cerebral peduncles, in middle cerebellar peduncles, and in thalamus, amygdala, and RLIC. Middle row: voxels located in thalamus, RLIC, PLIC, isthmus of the corpus callosum, and in right inferior parietal cortex, right supramarginal cortex, and left postcentral cortex. Right bottom: midsagittal view

Volumetric DTBM analysis showed smaller volumes in CP by voxel-wise differences (lower, more negative ln-Jac values), while no areas were detected in which CP had larger volumes than TD. Volumetric differences were pronounced in DGM structures, particularly in the thalamus, to a smaller extent in the globus pallidus, putamen, amygdala, hippocampus, and marginally in the caudate nucleus (Fig. 1). This is coinciding with the enlargement of the lateral ventricles in CP (see supplementary Fig. 1). We also observed clusters with voxel-wise differences in three cortical regions, indicating smaller volumes in CP within the right inferior parietal cortex, right supramarginal cortex, and left postcentral cortex. Smaller WM volumes were observed bilaterally in the PLIC, the RLIC, the cerebral peduncles, and the middle cerebellar peduncle. CP also had smaller volume of the isthmus of the corpus callosum, located in tracts connecting the left and right precuneus and the left and right superior parietal cortices. At tract-level, the voxels with smaller volume were found bilaterally in the area with overlapping tracts of medial lemniscus, corticospinal tract, and spinothalamic tract.

**Fig. 2:**
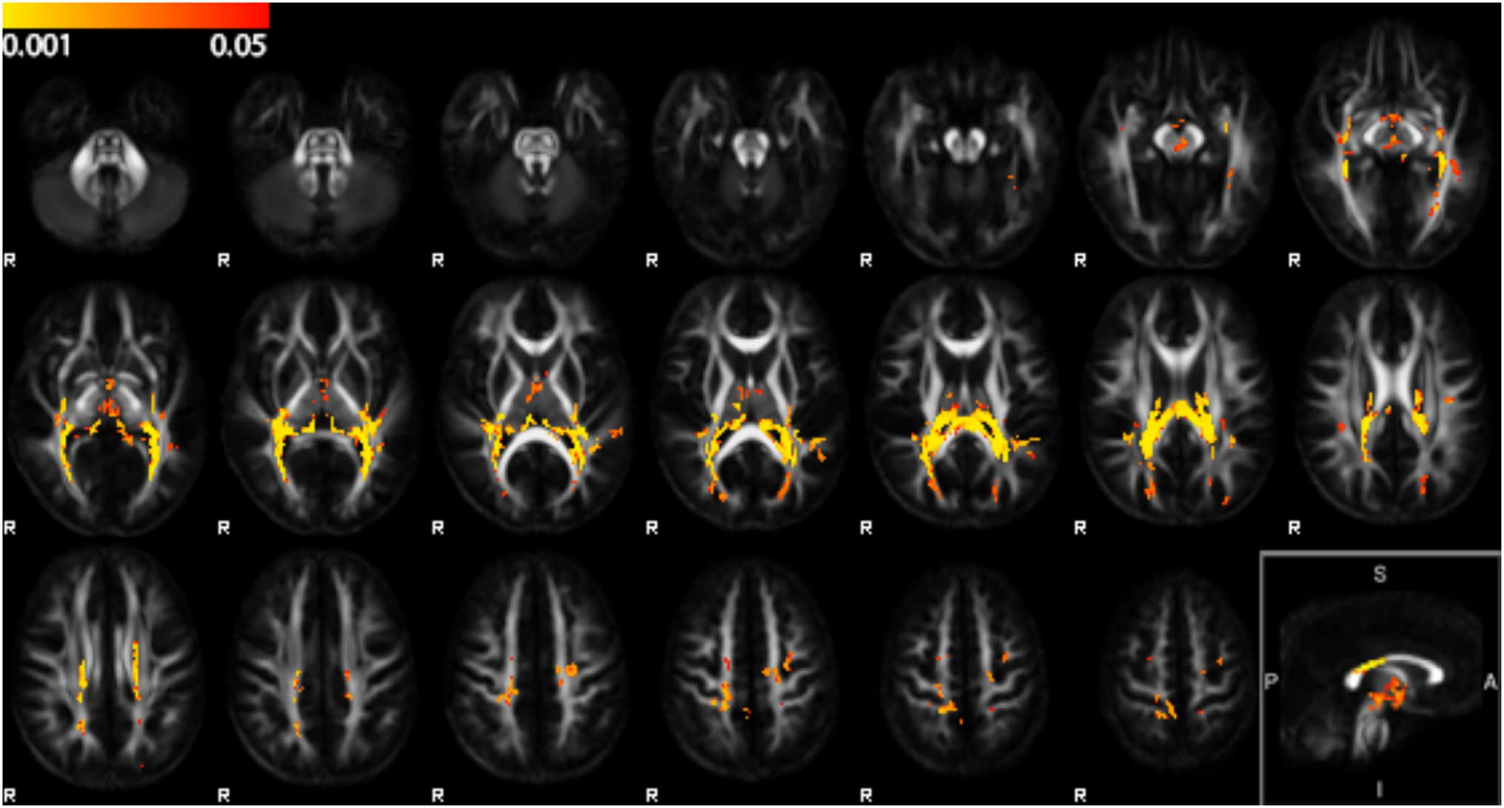
Voxel-wise FA comparison between CP and TD participants. Yellow–red clusters shown on top of the mean FA group template indicate areas with significantly lower FA in CP participants compared to TD participants (TFCE FWE-corrected p < 0.05, range of p-values indicated by the colorbar). Top row: voxels located in white matter tracts (such as posterior thalamic radiation). Middle row: voxels located in isthmus of corpus callosum, RLIC, and in white matter tracts. Lower row: voxels located in white matter tracts connecting the thalamus and superior parietal cortices. Right bottom: midsagittal view

Voxel-wise comparison of FA values between CP and TD at baseline revealed reduced FA in CP compared to TD, while there were no areas with increased FA in CP (Fig. 2). The RLIC bilaterally and the isthmus of the corpus callosum were the only areas in which both FA and volume were decreased. However, FA was reduced in a larger area of the corpus callosum, covering the whole isthmus, located in tracts between left and right postcentral and paracentral cortices, and in tracts connecting the thalamus and superior parietal cortices. Areas with reduced FA include the typical location of FLAIR hyperintensities due to periventricular leukomalacia (see supplementary Fig. 1). At the level of the brainstem, FA was reduced in tracts from thalamus to cerebellar cortex, but there were no FA differences within the cerebellum.

### Relationship between balance control and brain structure (objective 2)

Because the DTBM analysis showed more extended and spatially better localized differences between CP and TD than the structural T1-weighted scans, the relationship between balance control and brain structure was assessed within a ROI mask consisting of the combination of the volumetric and FA differences as shown in Figs. 1 and 2.

Voxel-wise correlation analysis with Challenge score for all participants at baseline showed large regions with positive correlation between Challenge score on the one hand and FA or volume on the other hand (at corrected p < 0.05). Those regions showed almost complete overlap with the differences in volume and FA observed in the comparison between CP and TD, and are therefore not shown. This means that the correlations within the whole group of participants at baseline were mainly driven by the large difference in Challenge score between CP and TD.

**Fig. 3:**
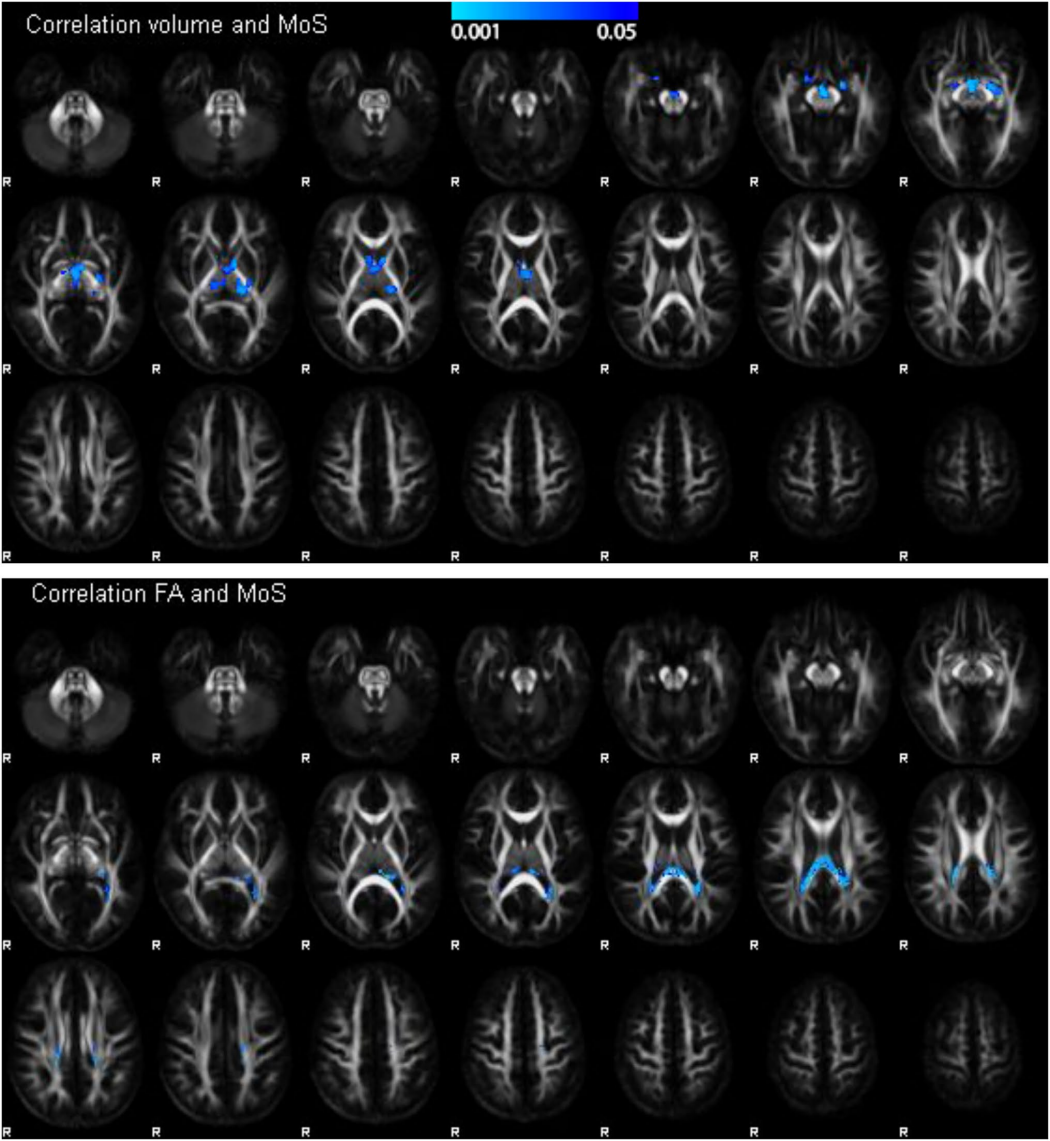
Voxel-wise correlation between volume and MoS (top) and between FA and MoS (bottom) for all participants at baseline. Blue–light-blue custers indicate areas with a significant negative correlation between volume or FA on the one hand and MoS score on the other hand (TFCE FWE-corrected p < 0.05, range of p-values indicated by the colorbar). Negative correlations with volume were observed in clusters within the thalamus and the amygdala. Negative correlations with FA were observed in the isthmus of the corpus callosum

Voxel-wise correlations with MoS for all participants at baseline were only negative, and spatially more limited than the volumetric or FA differences between CP and TD (Fig. 3). Negative correlations with volume were restricted to clusters within the thalamus and the amygdala, and negative correlations with FA were restricted to the isthmus of the corpus callosum. These correlations were observed within the whole group, but the effects were different between CP and TD, as shown in supplementary Fig. 2 for the correlation between volume in the thalamus or FA in the isthmus of the corpus callosum on the one hand and MoS on the other hand.

To remove driving effects of TD participants, we also performed voxel-wise correlation analysis within CP at baseline. In these analyses only single isolated pixels passed the significance threshold of corrected p < 0.05 threshold. When we evaluated results below the threshold of uncorrected p < 0.001 for exploratory reasons, we did not observe clustered correlations with the Challenge score. Clustered correlations with the MoS score below uncorrected p < 0.001 were only detected for volume, but these were positive, and located just within the ventricles, at the upper border of the thalamus. These findings could possibly suggest that larger ventricular volume correlated with higher MoS scores, but observations in CP children were variable (supplementary Fig. 2A). Also because voxel-based changes in CSF are difficult to interpret, the data do not support correlation with MoS score within the CP group.

### Effects of balance intervention (objective 3)

Total time played on the X-Box was 20.9 ± 5.0 h (range: 12.6–28.4 h), where 15 h were scheduled. For one child with CP, follow-up evaluation of balance control and MRI acquisition were arranged after four weeks instead of six weeks due to an upcoming surgery. However, within these four weeks, total time played of this child was over 15 h. For another child, total time played could not be read out due to loss of login details, but the parents indicated that the child had played for a sufficient time period.

At follow-up, mean Challenge score of children with CP had increased by 3.5 points (from 38.5 ± 13.1 points to 42.0 ± 13.3 points, p = 0.01). MoS values were not significantly altered (from 0.169 ± 0.030 m to 0.179 ± 0.043 m, p = 0.09).

**Fig. 4:**
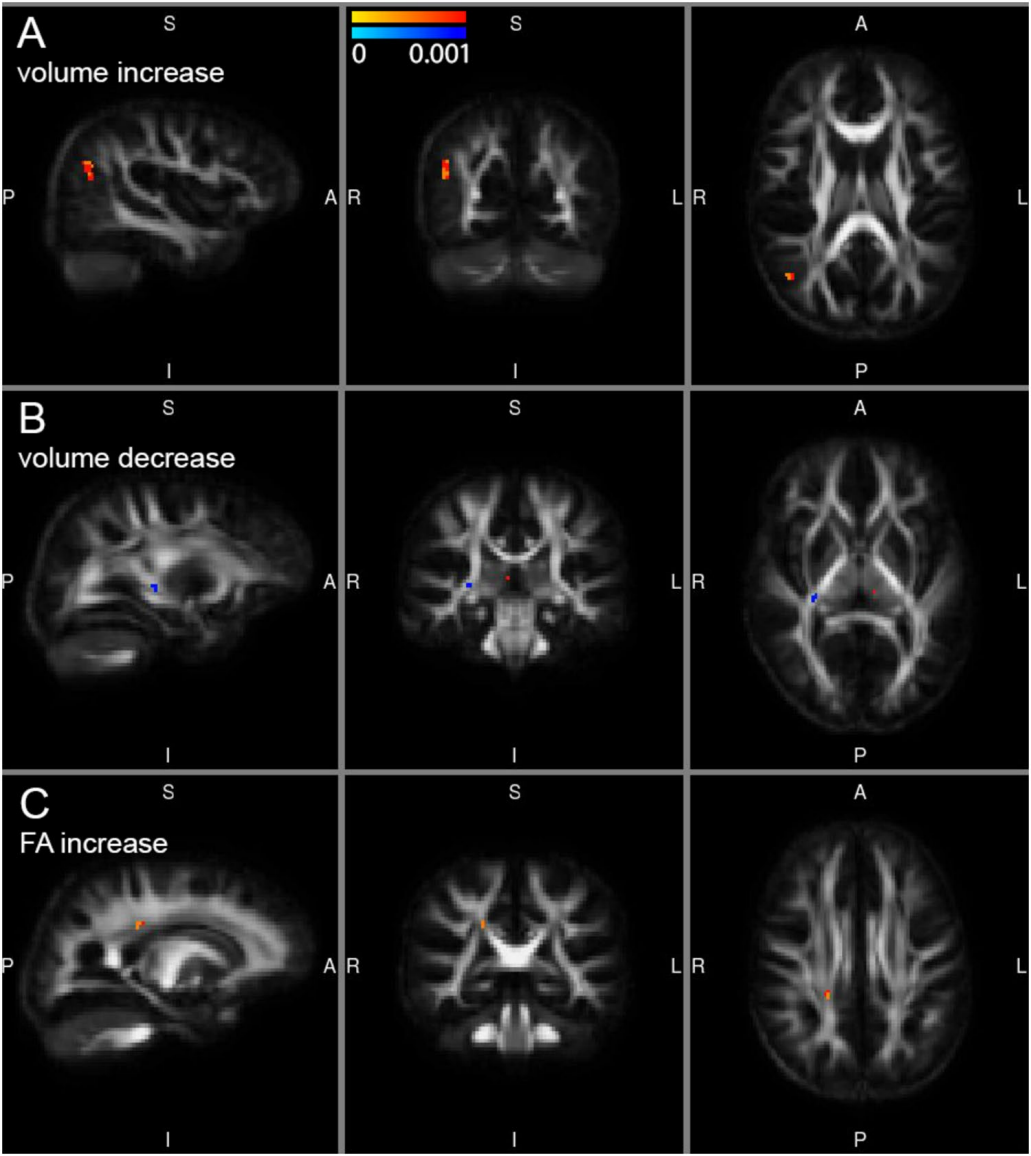
Voxel-wise longitudinal change in volume (**A**,** B**) and FA (**C**) in CP participants as an effect of balance training. **A** Yellow–red cluster (16 voxels) indicates an area with a trend for volume increase in the right inferior parietal cortex. **B** Blue–light-blue cluster (6 voxels) indicates an area with a trend for a volume decrease restricted to a few voxels in the right RLIC. **C** Yellow–red cluster (5 voxels) indicates an area in the right corticospinal tract with a trend for an increase of FA.Trends based on a threshold with TFCE, but FWE-uncorrected p < 0.001, range of p-values indicated by the color bars)

Using the strict threshold of TFCE-FWE-corrected p < 0.05, we did not observe voxel-wise changes in volume or FA in the longitudinal analysis within the ROI-mask defined before (based on areas with significant differences in either volume and/or FA between CP and TD). However, in the exploratory analysis we observed an indication for a volume increase in the right inferior parietal cortex, where a relatively large cluster was detected at the threshold of uncorrected p < 0.001 (corresponding to corrected p < 0.4) (Fig. 4A). In addition, a small cluster of voxels in the right RLIC indicated a volume decrease after balance training (visible at corrected p < 0.25) (Fig. 4B).

With regard to longitudinal changes in FA, we observed a few isolated voxels indicating either a decrease or an increase of FA, but we detected one small cluster within the right corticospinal tract (Fig. 4C) with a slightly stronger indication of increased FA after balance training (at uncorrected p < 0.001, visible at corrected p < 0.5).

**Fig. 5:**
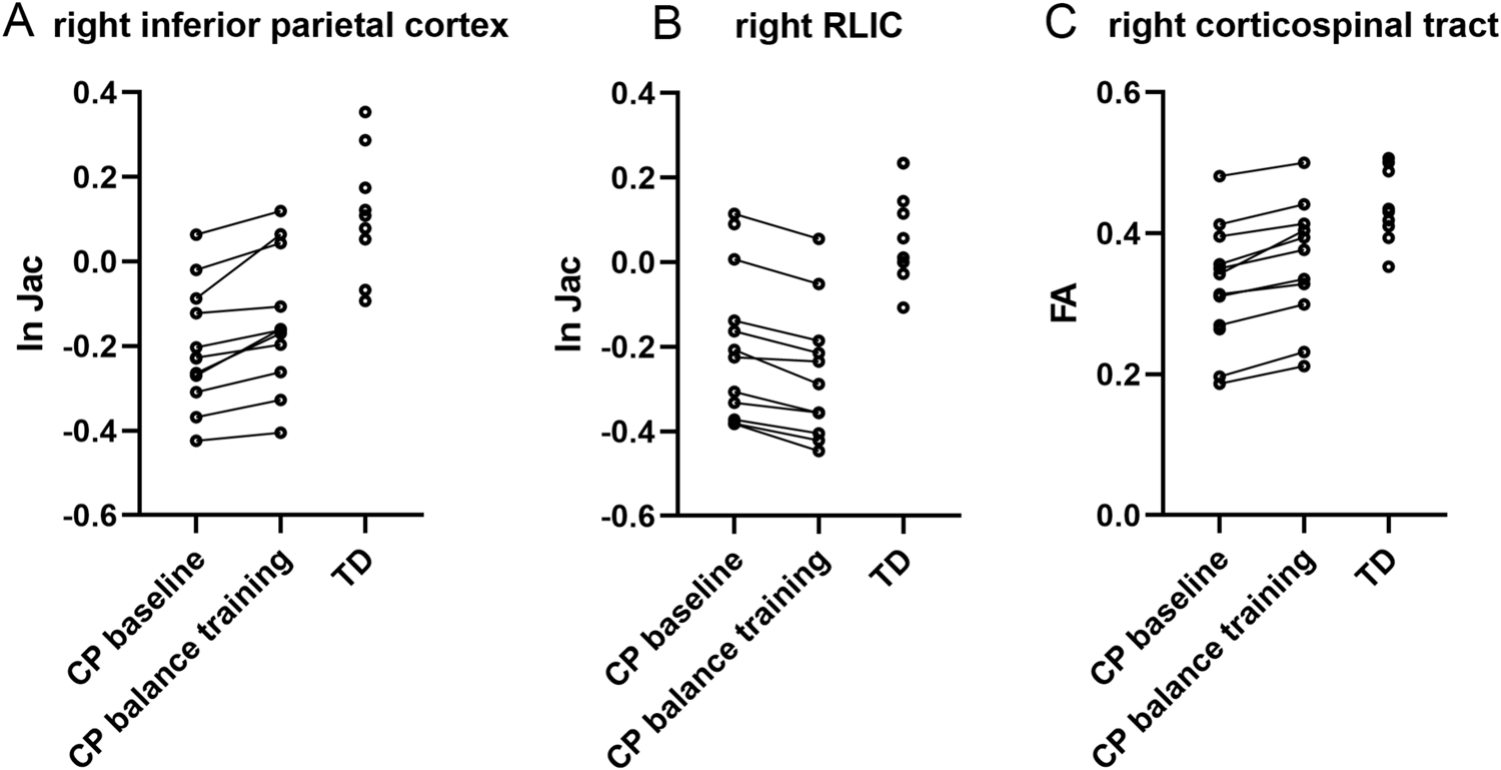
Visualization of the consistently increased volume in the right inferior parietal cortex (**A**) and increased FA in the right corticospinal tract (**C**) in CP participants as an effect of balance training. For comparison the values in TD children are shown as well. The consistent volume decrease in the small cluster in the right RLIC (**B**) was unexpected, considering the larger volume in the TD children. In **A** and **B** logarithmic Jacobian values (ln-Jac) are shown, which are defined with respect to the mean group template, where more negative values indicate a lower volume

To visualize the consistency of volume increase after balance training, we extracted the ln-Jac values from the cluster within the right inferior parietal cortex (16 voxels) for CP at baseline and after training, as well as for the TD participants at baseline (Fig. 5A). The difference between CP and TD was evident, as well as the increase in volume in all CP participants (towards TD values) as a result of balance intervention. (Note that logarithmic Jacobian values are defined with respect to the mean group template, where more negative values indicate a lower volume). The trend of decreased volume in the small cluster located in the right RLIC was also consistent, but the change was away from TD values (Fig. 5B). The FA increase in the cluster in the right corticospinal tract was consistent for all CP participants, and towards TD values (Fig. 5C).

## Discussion

The aim of this study was to improve our understanding of the structural brain correlates of balance control in children with CP. First, we showed that balance measures and various brain MRI measures differed between CP and TD. DTBM could confirm the differences in volume and showed differences in FA. DTBM was spatially sensitive in identifying volumetric differences within WM, DGM, and within several cortical areas. Second, we demonstrated that the relationship between volume or FA and Challenge scores were mainly driven by the differences in these balance measures between CP and TD groups. Third, after a 6-week balance training, we observed an improvement of clinical balance control. Although longitudinal changes in volume and FA did not pass a conservative statistical significance threshold, in an exploratory analysis we observed trends for an increase in FA of the right corticospinal tract, an increase in volume of the right inferior parietal cortex and a small volume decrease in the right RLIC, indicating that balance interventions have the potential to induce structural neuroplastic changes in the brain of children with CP.

Lower Challenge scores and higher MoS in the CP group confirmed that balance control was impaired compared to TD children, which confirms previous literature (Bruijn et al. 2013; Rethwilm et al. 2021; Jaatela et al. 2023). Also several volumetric and FA values were different between the groups. In our group of children with bilateral spastic CP, categorized as GMFCS II, volumes of WM were more affected than cortical GM, based on conventional MRI. The lower WM volume met expectations because most children had periventricular leukomalacia. In cerebral WM, we localized the regions with smaller volume and/or lower FA using DTBM. The internal capsule (both PLIC and RLIC) had a smaller volume, while the RLIC also had a lower FA. These results support the study of Nagae et al. (2007), who found, using a qualitative scoring system, that fibers of RLIC were abnormal more often than fibers of PLIC. The observation that not only PLIC but also RLIC, which contains the optic radiation, could be involved in CP pathophysiology is supported by the fact that cerebral visual impairment is common in these children (Philip et al. 2020). Also in our population 33% of the children had cerebral visual impairment, but in a post-hoc analysis we did not find an association between RLIC volume or FA and cerebral visual impairment (data not shown). Furthermore, we observed lower FA and smaller volume of the isthmus of the corpus callosum in CP, confirming findings in adolescents with CP (Jaatela et al. 2023). The isthmus contains tracts that bilaterally connect the precuneus, superior parietal cortices, postcentral, and paracentral cortices (Raybaud 2010), and microstructural changes of the isthmus could affect interhemispheric communication in CP (Weinstein et al. 2014; Hung et al. 2019). Using conventional T1-weighted MRI, we did not observe differences in total cerebellum volume, but we observed localized differences using DTBM, in particular a lower volume of the middle cerebellar peduncles in CP. At the level of the brainstem, FA was reduced in tracts from thalamus to cerebellar cortex, but there were no FA differences *within* the cerebellum. In contrast, Wang et al. (2014) described lower FA of middle and superior cerebellar peduncles in CP compared to TD, which correlated with general motor function in CP (covering the full functional mobility range (GMFCS I to V)). Since the children with CP in our study had relatively good motor function (GMFCS II), and mostly periventricular leukomalacia, the cerebellum may have been relatively unaffected. In accordance with existing literature (Scheck et al. 2014), volumes of hippocampal GM, basal ganglia and thalamus were substantially smaller in the CP group, as observed with conventional MRI and voxel-wise DTBM analysis. DTBM analysis indicated that several cortical areas were involved as well. Smaller volumes were localized within the right inferior parietal cortex, right supramarginal cortex and left postcentral cortex. These differences were not detected by cortical thickness measurements of conventional T1-weighted images, which summarize the thickness of relatively large cortical areas, and will be less sensitive to small, localized differences. To our knowledge, the localized cortical volume reductions have not been described before in CP, but all regions can be related to functions of the sensory-motor system, balance and proprioception. The inferior parietal cortex is involved in perception, planning and interpretation of sensory information (Battaglia-Mayer et al. 2006; Andersen 2011) and in human balance (Surgent et al. 2019). The supramarginal and postcentral cortex are part of the somatosensory association and primary somatosensory cortex, respectively. Both areas have been found to be important for proprioception (i.e. the perception of limbs or body segments in space or with respect to each other), and thus for postural control (Ben-Shabat et al. 2015; Kato and Izumiyama 2015; Qu et al. 2022; Steinberg et al. 2022).

Our next step was to relate the balance measures to MRI/DTI measures, and although we observed a few correlations within the whole group, these correlations were not evident within the CP group alone, similar to observations by Jaatela et al. (2023). A large area in the brain showed a positive correlation between Challenge scores for all participants at baseline and FA or volume (at corrected p < 0.05), suggesting better balance control with higher FA or volume in these regions. However, these correlations were mainly driven by the large difference in Challenge scores between the CP and TD groups. Within the CP group alone, we did not observe any correlations within brain tissue. The relation between balance and cerebral changes will be more complex than observed by the localized changes that can be recognized on a group level. Balance (and motor function in general) require structural and functional networks within the brain, and it may be expected that the networks of the children with CP in this study are differentially affected, although the majority had periventricular leukomalacia (Jacobs et al. 2023).

Challenge scores of children with CP significantly increased after the intervention, indicating improved balance control, coordination and speed. We also found trends for an increase in volume of the right inferior parietal cortex, in line with the function of this area in balance control (Surgent et al. 2019). The observed trend for an increased FA in a small region within the right corticospinal tract is according to our expectation, since higher FA is generally interpreted as improved WM integrity. Despite all limitations of this simplified interpretation (Jones et al. 2013; Vos et al. 2012), the FA values of all CP participants increased in the direction of values observed for TD children. In contrast, the trend for a decrease in volume of the right RLIC after balance training is puzzling. We speculate that WM reorganization has occurred, but this observation needs to be replicated in relation to balance intervention and in a larger sample.

Several strengths and limitations of this study can be specified. Above all, we want to highlight the importance of accurate registration in MRI/DTI studies in CP, considering the primary brain injuries. Therefore, the first strength of this study is the use of DTI-TK registration. To date, tensor-based registration is generally preferred over FA-based registration for DTI data (Bach et al. 2014; Wang et al. 2017). And although tensor-based registration is particularly suitable for aligning WM structures, GM and DGM structures are co-registered. In fact, in the whole brain voxel-wise analysis we observed localized volume differences in three cortical areas which are implicated in somatosensory function. Visual accuracy of segmentation using DTI-TK registration was good, even for children who had widespread lesions or tissue loss, and therefore we expect that our results are minimally affected by registration errors. To further ensure good registration, we corrected for echo-planar imaging distortions using reference scans with reversed phase-encode blips. This is important for images obtained at 3T or higher field strengths, and especially for relatively small ROIs in regions prone for geometric distortion artefacts, such as cerebral peduncles and inferior and superior cerebellar peduncles. The second strength of this study is the normalization of T1-weighted MRI derived volume measures for head size. This should not be overlooked in studies that involve children with CP, because these types of studies usually have a substantial heterogeneity in age of the children and thus in head sizes (Nellhaus 1968), as also observed in our study, and because children with CP often have smaller heads compared to TD children (Cheong et al. 2008). Other strengths include the longitudinal study design and the voxel-wise analyses which also capture small localized areas. Finally, the majority of participants in this study had periventricular leukomalacia, and all had spastic CP and GMFCS level II. Therefore, we may consider our study sample (although small) representative for this subgroup of the CP population (Himmelman et al. 2021).

One limitation of this study is the coverage of the diffusion-weighted MRI scans, which did not always include the full cerebellum, as a result of which FA and volume of only the superior parts of inferior and middle cerebellar peduncles could be examined. A second limitation is the use of only one high b-value in our DWI acquisition, since multi-shell diffusion-weighted MRI generally leads to better accuracy of diffusion tractography due to better distinction of crossing fibers. At the time of this study, multi-shell diffusion-weighted MRI was lengthy and considered not feasible for children with CP because of their difficulties with laying still. A third limitation is the absence of a gold standard for balance assessment in CP. Such a gold standard has not been defined in the literature. It is not known whether the Challenge score and the Margin-of-Stability completely represent the clinical balance performance. A fourth limitation is that it is not possible to disentangle the effect of the VR balance training on more general motor control versus pure balance control. Active training for balance control inherently uses motor actions, and changes in relation to the balance training may also be (partly) related to coordination improvements. Further, the control group did not participate in the training and did not receive the second MRI exam. Finally, the group of included children with CP was small, and we performed voxel-wise analyses in relatively large masks with a large number of voxels, as a result of which we needed to use a less conservative threshold for the exploratory analyses performed in the correlation analyses with balance measures and in the longitudinal analyses. In future, one could focus on these areas and select these a priori.

This study contributed to the unravelling of the structural brain correlates of balance control in children with bilateral spastic CP. We observed lower FA and smaller volumes of several brain structures including cortical areas involved in somatosensory function (i.e. areas involved in interpretation and integration of proprioception) in CP compared to TD. Correlations with balance measures were not evident. The inclusion of children with CP with a wider spectrum of motor dysfunction may be necessary to demonstrate neural correlates of balance in cross-sectional studies. Finally, the training resulted in improved balance control of children with CP, which was accompanied by subtle but consistent changes of highly localized areas in WM (right corticospinal tract and RLIC) and cortex (inferior parietal cortex). The findings of this study can guide future research on establishing individualized treatment possibilities for balance control in CP based on a person’s structural abnormalities, and how these abnormalities may predict therapy outcome. Furthermore, this study suggests that commercially available non-immersive gaming systems (like X-box and Kinect) could offer a cost-effective, engaging platform to promote neuroplasticity and improve balance in children with CP, warranting larger-scale trials. 

## Supplementary Information

Below is the link to the electronic supplementary material.

Supplementary file1 (PDF 1271 kb)

## Data Availability

The data generated and analysed during the current study are not publicly available due to General Data Protection Regulation, but are available from the corresponding author on reasonable request.

## Abbreviations

CP: Cerebral palsy
DTI: Diffusion tensor imaging
DGM: Deep grey matter
DTBM: DTI-based voxel-based morphometry
FA: Fractional anisotropy
GM: Grey matter
GMFCS: Gross Motor Function Classification System
GMFM: Gross Motor Function Measure
IIT: Illinois Institute of Technology
Ln-Jac: Natural logarithm of the Jacobian field
MNI: Montreal Neurological Institute
MoS: Medio-lateral margin of stability
PLIC: Posterior limb of internal capsule
RLIC: Retrolenticular limb of internal capsule
TBI: Traumatic brain injury
TD: Typically developing
WM: White matter
