# Supplementary material for: Structural brain correlates of balance control in children with cerebral palsy: baseline correlations and effects of training
Source: Brain Struct Funct. 2025 May 20;230(5):67. doi: 10.1007/s00429-025-02937-1 (PMC12092486; doi:10.1007/s00429-025-02937-1)
Supplement: Supplementary file 1 — Supplementary file1 (PDF 1271 kb) [file 429_2025_2937_MOESM1_ESM.pdf]

Supplementary text:

2.1 Study outline

The study was conducted as a part of the registered trial CP-RehOP (trial number: NTR6034/NL5854). The primary goal was to determine the effect of virtual reality balance training on postural and gait balance control in children with CP. Results and sample size considerations have been published (Meyns et al. 2022). Secondary goals were to examine the structural and functional brain networks involved in balance control in CP, and to investigate whether advances in balance control are supported by neuroplastic changes (https://cordis.europa.eu/project/id/660458).

2.4 Balance measures

MoS is a measure of balance control during steady-state gait. Balance control in humans is often described by the 'inverted pendulum' model (Hof, 2005, 2007). In such case, in static conditions, the vertical projection of the center of mass (CoM) needs to stay within the boundaries of the base of support to maintain stable. In dynamic conditions such as walking, however, one must account for the velocity of the center of mass as well, given that the CoM is moving already (i.e. the extrapolated center of mass or XCoM). The distance between the XCoM and the boundaries of the BoS was proposed as a measure of dynamic balance control (i.e. MoS) (Hof, 2008). As such, MoS was determined during walking on an instrumented treadmill. Prior to the measurements, body mass, body length and limb lengths were obtained, and a total of 39 retroreflective markers were attached to the children in accordance with the Vicon full body plug-in gait model (as implemented in Vicon Nexus 2.5). Children walked for 6 minutes at their preferred speed on the treadmill for habituation. Subsequently, children walked at preferred walking speed during one trial of one minute, during which kinetic and kinematic data was collected. The position of the XCoM was calculated to correct for the effects of walking speed on the position of the CoM (Hof 2005). XCoM was defined as CoM, plus its velocity times a factor: √(CoM height/acceleration of gravity). MoS was defined as the minimal medio-lateral distance between the XCoM and the lateral malleolus of the leading foot (Hof et al. 2005; Hak et al. 2013). Average MoS was calculated as the average value over all available steps.

References:

Hak L, Houdijk H, Steenbrink F, et al (2013) Stepping strategies for regulating gait adaptability and stability. Journal of Biomechanics 46:905–911. https://doi.org/10.1016/j.jbiomech.2012.12.017

Hof AL (2005) Comparison of three methods to estimate the center of mass during balance assessment. J. Biomech. 38, 2134–2135. https://doi.org/10.1016/j.jbiomech.2005.03.029

Hof AL, Gazendam MGJ, Sinke WE (2005) The condition for dynamic stability. Journal of Biomechanics 38:1–8. https://doi.org/10.1016/j.jbiomech.2004.03.025

Hof AL (2007) The equations of motion for a standing human reveal three mechanisms for balance. J. Biomech. 40, 451–457. doi: 10.1016/j.jbiomech.2005.12.016

Hof AL (2008) The 'extrapolated center of mass' concept suggests a simple control of balance in walking. Hum. Mov. Sci. 27, 112–125. doi: 10.1016/j.humov.2007.08.003

Meyns P, Blanckaert I, Bras C, et al (2022) Exergaming improves balance in children with spastic cerebral palsy with low balance performance: results from a multicenter controlled trial. Disability and Rehabilitation 44:5990–5999. https://doi.org/10.1080/09638288.2021.1954704

Supplementary Table 1: Primary brain injuries of children with CP

Children with CP	Primary brain injury
01	Periventricular leukomalacia
02	Periventricular leukomalacia
03	Periventricular leukomalacia
04	Periventricular leukomalacia, damage to left thalamus, motor cortex atrophy
05	Periventricular leukomalacia
06	Periventricular leukomalacia
07	Periventricular leukomalacia
08	Periventricular leukomalacia
09	Tissue loss due to bleeding in left temporal area, damage to bilateral putamen and thalamus
10	Left arteria cerebri media infarct, superficial watershed infarcts (left>right)
11	Periventricular leukomalacia
12	Periventricular leukomalacia

Abbreviations: CP: cerebral palsy

Supplementary Fig. 1: FLAIR images of all CP participants

CP01 – periventricular leukomalacia

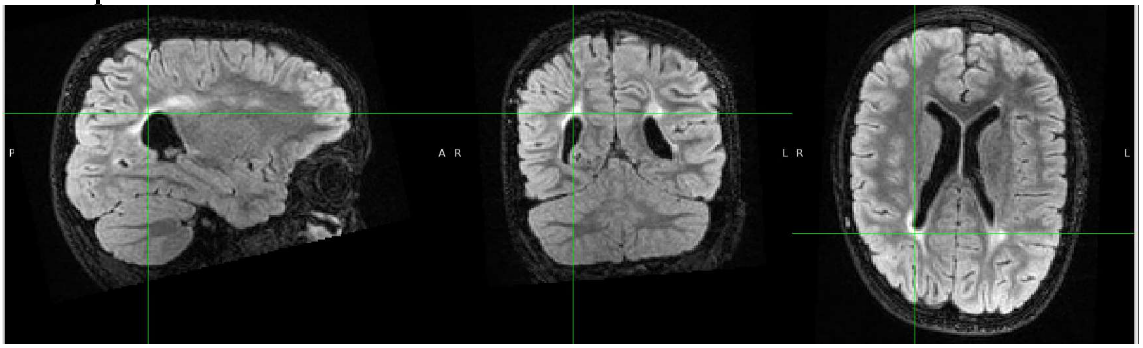

CP02 – periventricular leukomalacia

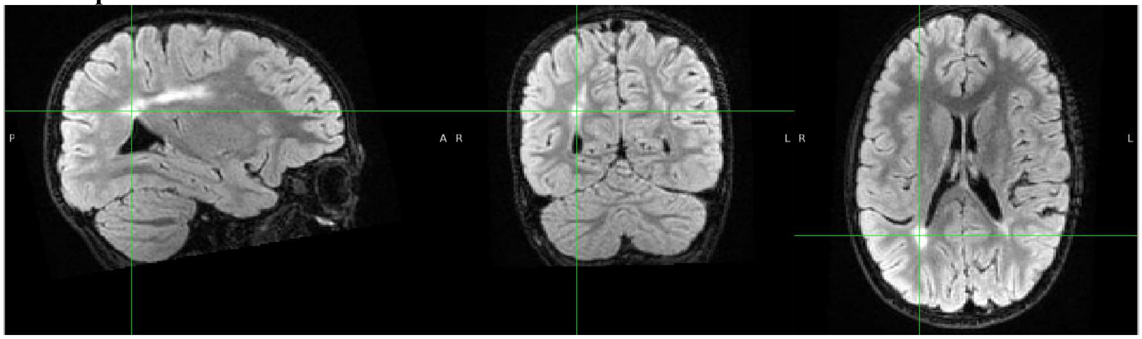

CP03 – periventricular leukomalacia

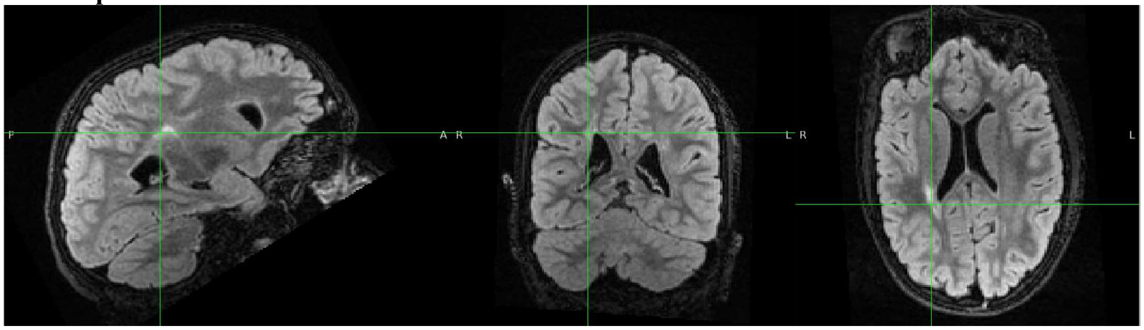

CP04 – periventricular leukomalacia – damage to left thalamus, motor cortex atrophy

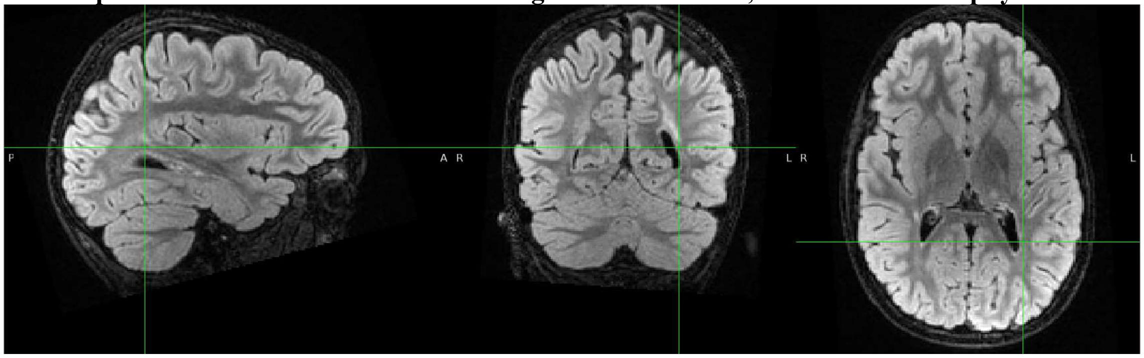

CP05 – periventricular leukomalacia

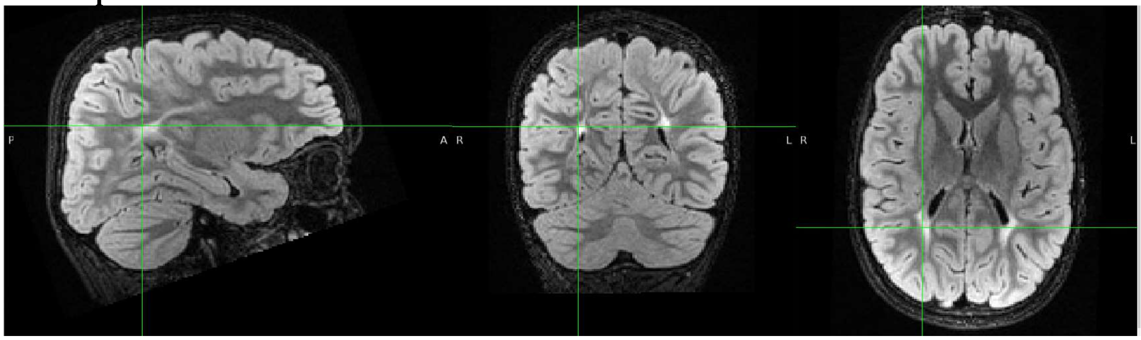

CP06 – periventricular leukomalacia

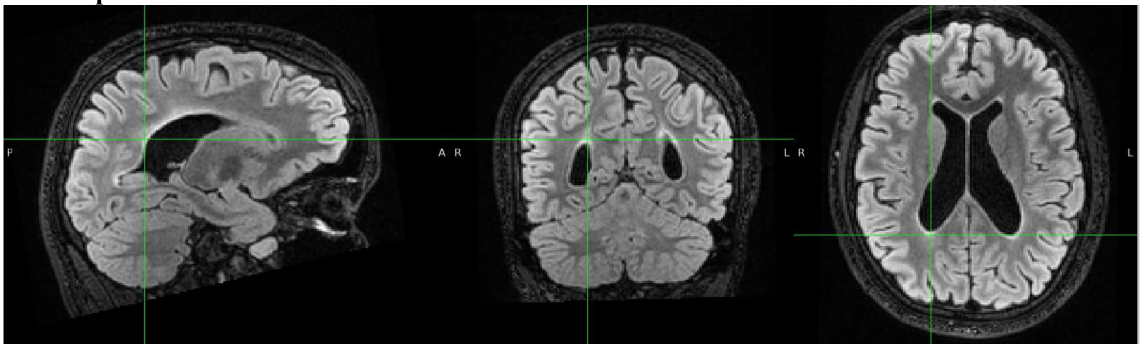

CP07 – periventricular leukomalacia

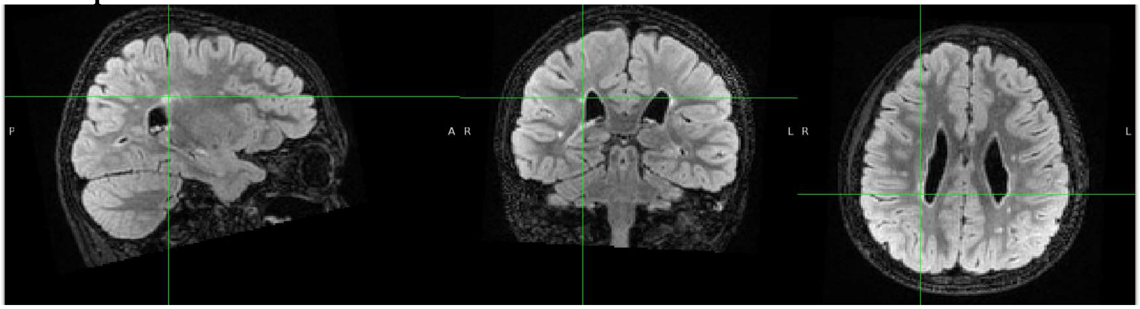

CP08 – periventricular leukomalacia

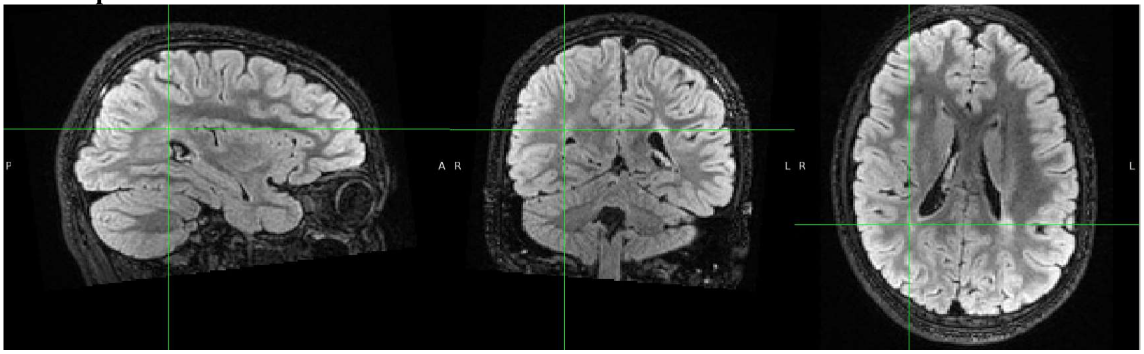

CP09 – tissue loss due to bleeding in left temporal area, damage to bilateral putamen and thalamus

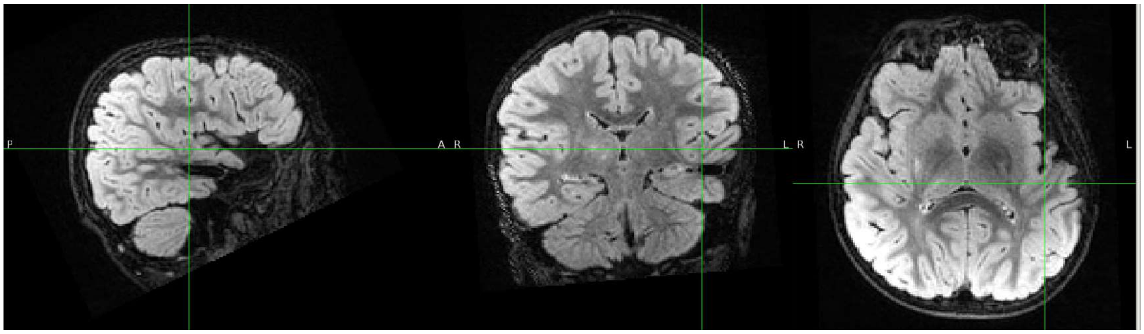

CP10 – Left arteria cerebri media infarct, superficial watershed infarcts (left>right)

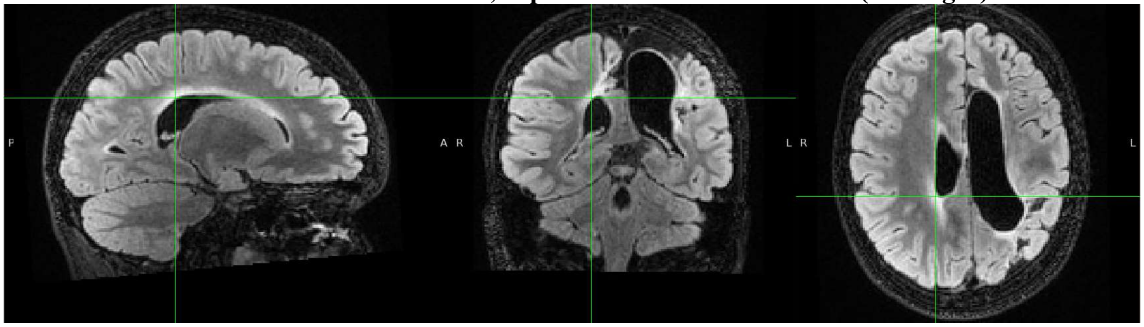

CP11 – periventricular leukomalacia

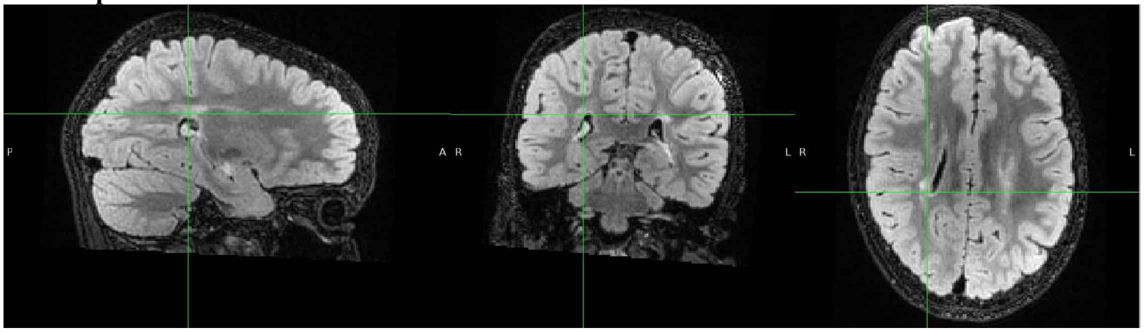

CP12 – periventricular leukomalacia

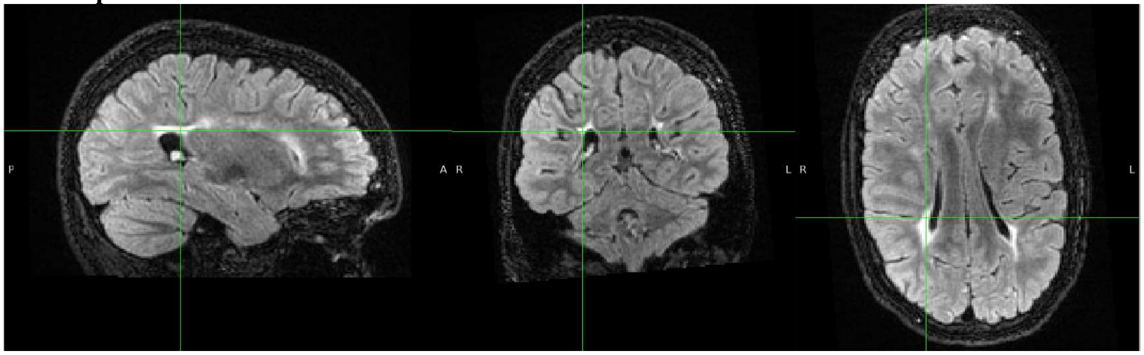

Supplementary Fig. 2:

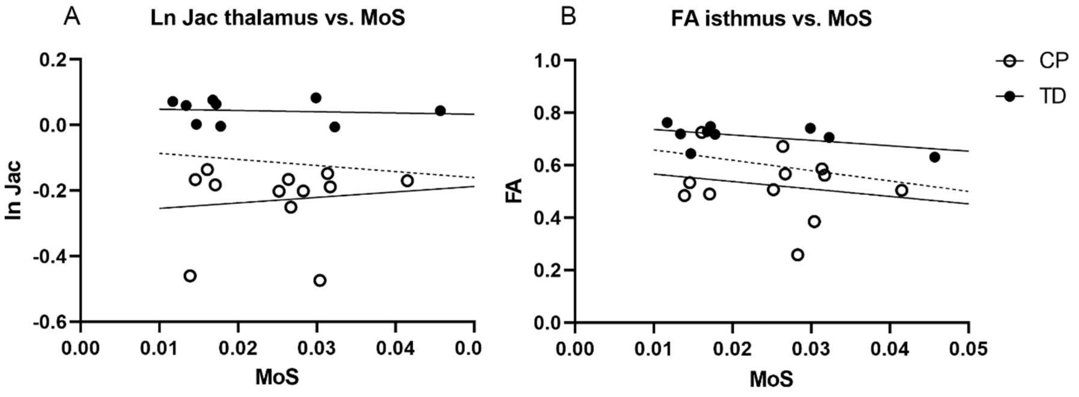

Supplementary Fig. 2: Visualization of the correlation between (A) volume (ln-Jac) in the thalamus vs. MoS and (B) between FA in the isthmus of the corpus callosum vs. MoS, indicated for CP participants (open circles) and TD participants (closed circles) Although correlations are significant within the whole group, indicated with the dotted line, there is no correlation within the CP group alone. Moreover, the variability between the CP participants is relatively large.
